# Role of nutritional vitamin D in chronic kidney disease-mineral and bone disorder: A narrative review

**DOI:** 10.1097/MD.0000000000033477

**Published:** 2022-04-07

**Authors:** Yingjing Shen

**Affiliations:** a Department of Nephrology, Shanghai Tianyou Hospital, Shanghai, China.

**Keywords:** chronic kidney disease, nutritional vitamin D, vascular calcification, Wnt signaling pathway

## Abstract

Chronic kidney disease-mineral and bone disorder has complex and diverse clinical manifestations, including the simplest abnormalities of calcium, phosphorus and parathyroid hormone detected in blood, abnormalities of bone transformation and mineralization in bone, and calcification of blood vessels or other soft tissues detected on imaging. Patients with CKD-MBD combined low bone mineral density and fragility fractures are referred to as CKD-MBD with low bone mineral density. Vascular calcification refers to ectopic deposition of calcium phosphate in the blood vessel walls and heart valves. The degree of vascular calcification was inversely proportional to bone mineral density. The more severe the degree of vascular calcification, the lower the bone mineral density, and the higher the risk of death, indicating that the bone-vascular axis exists. Activation and alteration of the Wnt signaling pathway are central to the treatment of vascular diseases in uremia. Vitamin D supplementation can prevent secondary hyperparathyroidism, activate osteoblasts, relieve muscle weakness and myalgia, and reduce vascular calcification. Nutritional vitamin D may improve vascular calcification in uremia patients by regulating Wnt signaling pathway.

## 1. Introduction

Chronic kidney disease-mineral and bone disorder or CKD-MBD, is a syndrome involving mineral and bone metabolism disorders caused by chronic kidney disease. One or more of the following clinical manifestations: abnormal metabolism of calcium, phosphorus, parathyroid hormone, and vitamin D; abnormal bone turnover; mineralization, bone mass, linear bone growth, bone strength; vascular or other soft tissue calcification.^[1]^ Osteoporosis is a systemic metabolic bone disease characterized by low bone mass and destruction of the bone microstructure, resulting in increased fragility and susceptibility to fractures. Both diseases can seriously affect bone metabolism and increase the risk of fracture; therefore, there may be a close relationship between them.

The incidence of osteoporosis in patients with chronic kidney disease is higher than that in the general population.^[2,3]^ The prevalence of osteoporosis varied with the stage of CKD, and increased with decline in renal function (<0.05).^[4,5]^ Fractures in patients with early CKD are more similar to traditional osteoporosis than to CKD-MBD.^[6]^ Most patients with CKD4-5 have a decreased ability of the kidney to regulate minerals, so there is a widespread manifestation of decreased bone mineral density or varying degrees of CKD-MBD.^[7]^ Patients with CKD stage 3B and above who have higher bone marrow fat content and lower bone mineral density are prone to vertebral fracture. It may be related to the involvement of osteosclerostin protein.^[8]^ According to the 2009 Kidney Disease: Improving Global Outcomes guidelines, CKD-MBD patients with stage 3 to 5 CKD who have decreased bone mineral density (BMD) and fragility fractures should be referred to as CKD-MBD with low BMD. Patients with CKD have a high prevalence of osteoporosis and incidence of fractures, especially hip fractures, and an increase in final morbidity and mortality rates.^[9]^ For patients with multiple risk factors, monitoring should be strengthened to prevent the falls and fractures.^[10]^

Vascular calcification (VC) is the ectopic deposition of calcium phosphate in the walls of blood vessels and heart valves, including the coronary arteries, abdominal aorta, iliac arteries, and femoral arteries.^[11]^ Previous studies have suggested that VC formation is a passive process, mediated by cell death and hydroxyapatite mineral infiltration in blood vessels. However, many recent studies have shown that VC is an active process in bone formation, and the key is the phenotypic transformation of vascular smooth muscle cells.^[12]^ Activated fusiform vascular cells can be transformed into spherical osteoblast-like cells and biomineralization of the extracellular matrix is induced.^[13]^ The pathology is generally divided into 2 categories: intimal and medial. The former is related to lipid and cholesterol deposition, which damages the endothelial cells and causes atherosclerosis. The latter is induced by mineral deposition in vascular smooth muscle cells.^[14]^ Vascular calcification in CKD rats is mainly due to the deposition of hydroxyapatite crystals in the vascular media.^[15]^ Endothelial cell-derived exosomal miR-670-3p can promote arterial calcification by targeting insulin-like growth factor-1, which may be a potential therapeutic target for arterial calcification in hemodialysis patients.^[16]^

There is a close relationship between vascular calcification and osteoporosis in CKD patients.^[17]^ Vascular smooth muscle cells and bone morphogenetic cells are derived from the same mesenchymal stem cell line. During embryogenesis, bone-derived and vascular-derived factors interact with each other, and eventually bone and blood vessels develop simultaneously.^[18]^ A recent study on patients with CKD highlighted the important clinical relationship between osteoporosis and vascular calcification. They found a negative correlation between aortic calcification, high ankle-brachial ratio, and BMD, and that the BMD of the femur and femoral neck and total BMD were associated with important lesions of the heart valve.^[19]^ Cardiac valve calcification and aortic calcification in patients with CKD are associated with cardiovascular disease, and the more severe the calcification, the higher is the risk of death.^[20]^ Arterial calcification at different locations is associated with different levels of mortality risk, and lateral abdominal radiography may not be superior to other radiographs in predicting patient outcomes.^[20]^ Patients with CKD experience either cortical or trabecular bone loss over time, but not both. The progression of vascular calcification in patients with CKD is associated with chronic trabecular bone loss, but not cortical bone loss.^[21]^ Maria L Mace established a new animal model by transplanting the severely calcified aortas of uremic rats into healthy rats. It was found that the production of osteopontin, a bone mineralization inhibitor, was reduced in aortic transplant recipient rats, osteoblasts were reduced in bone tissue morphology, and the mineral density of bone trabecular tissue was lower.^[22]^ Vascular calcification acts on bone through the Wnt signaling pathway, which induces bone loss, reduces bone density, and destroys bone metabolism, which indicates that there is an interaction between blood vessels and bone tissue.^[22]^

The Wnt signaling pathway is a classical signaling pathway involved in bone metabolism. Wnt glycoprotein is an extracellular signaling ligand that can stabilize the intracellular transcription factor β-catenin after activation and further stimulate the production of osteoblast factors such as Runx2 and osterix, thus increasing the number of bone cells and decreasing the number of osteoclasts, resulting in enhanced bone formation and decreased bone resorption.^[23,24]^ Sclerostin is a protein encoded by sclerostin gene secreted by bone cells, which can inhibit the transmission of Wnt signaling pathway. In renal failure, serum osteosclerotic protein levels increase, thereby reducing bone formation.^[25]^ The Wnt signaling pathway is activated during hyperphosphate-induced vascular calcification.^[26]^ The role of sclerostin in vascular calcification is inconsistent.^[27]^ Studies have shown that the expression of osteosclerostin protein is enhanced in calcified vascular smooth muscle cells, and the expression of its messenger ribonucleic acid (RNA) is significantly up-regulated in skin tissues with calcification defenses and calcified aortic valves.^[28]^ However, some studies have suggested that osteosclerostin has a protective effect against vascular calcification.^[29]^ Disorder and alteration of the Wnt signaling pathway is the core of vascular and bone diseases in uremia.^[18,27]^

Currently, in clinical practice, CKD-MBD treatment is limited to 3 indicators: serum calcium, phosphorus, and parathyroid hormone (PTH). With the irrational use of active vitamin D becoming increasingly common, side effects such as hypercalcemia, vascular calcification, and low transformation bone disease have become increasingly prominent.^[30]^ Aortic calcification scores were higher in patients with nondynamic bone disease and more severe aortic sclerosis.^[5]^ The application of new drugs such as lanthanum carbonate, Siviram and Cinacalcet, causes calcium, phosphorus and PTH to fluctuate and bone lesions to become more complex. In recent years, there have been many studies on the use of anti-osteoporosis drugs, such as bisphosphonates, calcitonin and teriparatide in the treatment of chronic kidney disease, but the use of these drugs is still limited.

Regarding vascular calcification, the 2017 Kidney Disease: Improving Global Outcomes CKD-MBD guidelines recommend that patients with CKD who are not on dialysis avoid supplementation with calcitriol and vitamin D analogs because excessive supplementation leads to hypercalcemia and hyperphosphatemia, which may promote vascular calcification.^[31]^ If treatment is initiated for severe and progressive hyperparathyroidism, calcitriol or an active vitamin D analogue should be started at a low dose and then titrated based on the parathyroid hormone response.^[31]^ Calcitriol and paricalcitol have different effects on vascular calcification, associated with differential modulation of the Wnt/β-catenin pathway. Calcitriol activates the Wnt/β-catenin pathway, up-regulates the expression of osteogenic markers such as BMP2, Ruru2, Msx2, and osteocalcin, and increases calcification, whereas paricalcitol down-regulates Wnt/β-catenin signaling and osteogenic marker expression and decreases calcification.^[26]^

Nutritional vitamin D (NVD) is an important trace element in the human body. It is a complex organic molecule extracted from cholesterol and plays a key role in maintaining the physiological balance of blood calcium.^[32]^ It is available in 2 separate fat-soluble forms: ergocalciferol (vitamin D2) and cholecalciferol (vitamin D3).^[3]^ Vitamin D3 is the main form of vitamin D.^[33]^ Because in addition to dietary vitamin D, the skin of mammals synthesizes physiological and endogenous vitamin D3 from 7-dehydrocholesterol after exposure to ultraviolet light.^[32,34]^ In plants, vitamin D2 is formed by ultraviolet radiation.^[3,35]^ The sum of the 2 is what we usually call 25-hydroxyvitamin D (25 (OH) D), which measures the metabolic status of vitamin D. Most clinicians refer to the recommendations of the Endocrine Society, which define a 25 (OH) D concentration of < 20 ng/mL as deficient, a 25 (OH) D concentration of 21 to 29 ng/mL as deficient, and a 25 (OH) D concentration of > 30 ng/mL as normal.^[36,37]^ The morbidity associated with NVD deficiency is high. 37% of the world’s population has a 25 (OH) D level below 20 ng/mL, and 6.7% of the population has a 25 (OH) D level below 10 ng/mL. The average per capita 25 (OH) D is less than 30 ng/mL (27.9 ng/mL), which has become a global human health problem.^[33]^

As we all know, one very important physiological role of nutritional vitamin D is to achieve the balance of mineral-bone metabolism by regulating the homeostasis of calcium. Vitamin D deficiency is also related to the development or deterioration of extraosseous diseases, such as skin, respiratory, endocrine, urinary, cardiovascular, and neurodegenerative diseases.^[33,38]^ 25 (OH) D can modulate the activity of innate and adaptive immune responses by acting on vitamin D receptors. Vitamin D can affect the lungs and immune system during the fetal and neonatal periods, and 25 (OH) D deficiency occurs during pregnancy and increases the incidence of asthma in young children after delivery.^[39]^

As recommended in clinical guidelines for vitamin D management, vitamin D3 appears to be the most logical choice for patients with vitamin D deficiency who require a precisely controlled equivalent of additional vitamin D supplementation, possibly because of its greater lipophilicity and slower pharmacokinetic clearance.^[33]^ Compared with intramuscular injection, the blood level of 25 (OH) D increased by 3 times after oral administration for 1 month.^[38]^ Each individual’s response to vitamin D3 supplementation differ and can be classified as high, medium, or low responders. Low vitamin D responders are most sensitive to vitamin D deficiency and require a higher daily dose of vitamin D3 (about 50–100 µg) to maintain optimal endocrine activity of vitamin D.^[39]^ Vitamin D receptor gene polymorphisms affect individual susceptibility to osteoporosis and the response to vitamin D supplementation. 25 (OH) D had a significant dose-dependent relationship with the rs1544410, rs731236, and rs11568820 genotypes. In particular, the rs731236 mutation genotype A/A of the vitamin D receptor gene is strongly associated with 25 (OH) D deficiency.^[38]^ Most studies of vitamin D supplementation have used doses of 400–1000 IU/d. Low doses of vitamin D are safe, however, taking >4000 IU per day can cause more falls and fractures.^[32]^

Serum 25 (OH) D levels in patients with CKD begins to decrease during the second stage of CKD.^[40]^ In the late stage of CKD, vitamin D deficiency is caused by impaired skin synthesis of vitamin D3, strict dietary control and reduced sunshine.^[30,41]^ The parathyroid glands become larger, calcium-sensing receptors and vitamin D receptors decrease, and enzymes that produce vitamin D increase, keeping vitamin D in a state of starvation.^[42]^ High-dose daily vitamin D3 (8000 IU/d) has been shown to prevent secondary hyperparathyroidism in patients with stage 3 to 4 CKD without increasing the risk of hypercalcemia and hyperphosphatemia^[37,43]^ Vitamin D2 also inhibited the increase of PTH level in CKD3-4 patients.^[44]^ NVD supplement therapy is only effective when the blood 25 (OH) D level is less than 30 ng/mL. It can correct hyperparathyroidism, restore blood calcium and phosphorus level to normal, reduce bone transport, and improve bone mineral density.^[32,45]^ When the serum 25 (OH) D level was more than 30 ng/mL, PTH tended to be stable.^[40]^ Compared to active vitamin D, NVD can activate osteoblasts, maintain the level of 25 (OH) D, and relieve muscle weakness and myalgia.^[46]^ It is of great significance in the treatment of osteoporosis.^[41,47]^ NVD not only plays a key role in bone metabolism, but also improves bone remodeling caused by osteoblasts.^[48,49]^ NVD supplementation can affect the vascular system, reduce the risk factors of vascular calcification, and alleviate the degree of vascular calcification.^[50]^

A randomized controlled study in 2021 enrolled 60 patients undergoing maintenance hemodialysis. The treatment group received 200 IU of vitamin D3 per month, and blood levels of 25 (OH) D and fetuin-a increased significantly after 3 months.^[51]^ The best cutoff values of serum 1,25 (OH)_2_ D levels for predicting aortic and mitral valve calcification were ≤ 12.5 pg/dL (sensitivity 80.8%, specificity 70.0%) and ≤ 11.9 pg/dL (sensitivity 71.6%, specificity 70.8%), respectively, using receiver operating characteristic curve analysis. 1,25 (OH)_2_ D deficiency is independently associated with aortic and mitral calcifications, suggesting that serum 1,25 (OH)_2_ D levels may be a potential biomarker of aortic and mitral calcifications in these patients.^[49]^ Vitamin D3 supplementation has been shown to treat vitamin D deficiency and increase fetuin-α levels in dialysis patients.^[51]^ Pulse wave velocity may also be reduced.^[52]^ Multivariate logistic regression analysis showed that 25 (OH) D level (odds ratio [OR]: 0.895, 95% confidence interval [CI] 0.828–0.968, *P* = .005) and age (OR: 1.140, 95% CI 1.088–1.194, *P* < .001) were an independent risk factors for peripheral arteriosclerosis in patients with CKD3-5. The lower the serum 25 (OH) D level and the older the age, the more likely it is to lead to atherosclerosis.^[53]^

In summary, the Wnt signaling pathway may be an important mechanism for the onset of CKD-MBD, and nutritional vitamin D may play an important role in its treatment.

## Author contributions

**Conceptualization:** Yingjing Shen.

**Formal analysis:** Yingjing Shen.

**Investigation:** Yingjing Shen.

**Methodology:** Yingjing Shen.

**Resources:** Yingjing Shen.

**Supervision:** Yingjing Shen.

**Validation:** Yingjing Shen.

**Writing – original draft:** Yingjing Shen.
